# Exploring the concept and management of post ictal psychosis through a clinical case and review

**DOI:** 10.1192/j.eurpsy.2023.1613

**Published:** 2023-07-19

**Authors:** A. Compaired Sánchez, B. Lázaro Alonso, J. Gómez-Arnau Ramírez

**Affiliations:** Psychiatry and Mental Health, Ramon y Cajal Universitary Hospital, Madrid, Spain

## Abstract

**Introduction:**

Postictal Psychosis of Epilepsy (PIPE), part of the group collectively known as Psychosis of Epilepsy, is characterized by an onset of confusion or psychotic symptoms within one week of apparent normal mental function. PIPE has been argued to be underdiagnosed in the clinical population, perhaps due to a failure to recognize the temporal relation between the seizure and the psychotic episode.

**Objectives:**

To explore the concept and management of post ictal psychosis.

**Methods:**

We present a clinical case and a review of the literature concerning post ictal psychosis.

**Results:**

We report the case of a 36 year old woman with focal refractory epilepsy after a likely episode of limbic encephalitis in 2015. Cognitive and psychiatric sequelae in the form of depressive symptoms, in treatment with neurology and psychiatry since 2021. One previous episode of psychotic symptoms during seizures. Worsening of seizure frequency since march of 2022 with apparent normalization (absence of seizures after dose reduction of eslicarbamazepine and introduction oflamotrigine) for about four days before being hospitalized in the neurology unit due tobehavioral abnormalities. During psychiatric exploration, the patient showed signs of partial clouding of consciousness with manierisms, ecopraxias and ecolalias; verbigerance in the form of the neurologist’s name and bizarre movements like looking behind suggestive of sensoroperceptive disturbances. The symptomatology resolved itself during the following week after treatment with diazepam.

Finally, a narrative review concerning the case was also performed; with particular emphasis on antipsychotic drugs with low risk of lowering seizure threshold (such as risperidone or aripiprazole) as the recommended treatment.

**Conclusions:**

Our findings point to the relevance of Postictal Psychosis of Epilepsy as a clinical entity. Further studies on pathogenic mechanisms and therapeutic management are required.

**Disclosure of Interest:**

None Declared

